# MicroRNA-330-5p as a Putative Modulator of Neoadjuvant Chemoradiotherapy Sensitivity in Oesophageal Adenocarcinoma

**DOI:** 10.1371/journal.pone.0134180

**Published:** 2015-07-29

**Authors:** Becky A. S. Bibby, John V. Reynolds, Stephen G. Maher

**Affiliations:** 1 School of Biological, Biomedical and Environmental Sciences, University of Hull, Hull, United States of America; 2 Department of Surgery, Institute of Molecular Medicine, Trinity College Dublin, St. James Hospital, Dublin, Ireland; Peking Union Medical College Hospital, CHINA

## Abstract

Oesophageal adenocarcinoma (OAC) is the sixth most common cause of cancer deaths worldwide, and the 5-year survival rate for patients diagnosed with the disease is approximately 17%. The standard of care for locally advanced disease is neoadjuvant chemotherapy or, more commonly, combined neoadjuvant chemoradiation therapy (neo-CRT) prior to surgery. Unfortunately, ~60-70% of patients will fail to respond to neo-CRT. Therefore, the identification of biomarkers indicative of patient response to treatment has significant clinical implications in the stratification of patient treatment. Furthermore, understanding the molecular mechanisms underpinning tumour response and resistance to neo-CRT will contribute towards the identification of novel therapeutic targets for enhancing OAC sensitivity to CRT. MicroRNAs (miRNA/miR) function to regulate gene and protein expression and play a causal role in cancer development and progression. MiRNAs have also been identified as modulators of key cellular pathways associated with resistance to CRT. Here, to identify miRNAs associated with resistance to CRT, pre-treatment diagnostic biopsy specimens from patients with OAC were analysed using miRNA-profiling arrays. In pre-treatment biopsies miR-330-5p was the most downregulated miRNA in patients who subsequently failed to respond to neo-CRT. The role of miR-330 as a potential modulator of tumour response and sensitivity to CRT in OAC was further investigated *in vitro*. Through vector-based overexpression the E2F1/p-AKT survival pathway, as previously described, was confirmed as a target of miR-330 regulation. However, miR-330-mediated alterations to the E2F1/p-AKT pathway were insufficient to significantly alter cellular sensitivity to chemotherapy (cisplatin and 5-flurouracil). In contrast, silencing of miR-330-5p enhanced, albeit subtly, cellular resistance to clinically relevant doses of radiation. This study highlights the need for further investigation into the potential of miR-330-5p as a predictive biomarker of patient sensitivity to neo-CRT and as a novel therapeutic target for manipulating cellular sensitivity to neo-CRT in patients with OAC.

## Introduction

Globally, oesophageal adenocarcinoma (OAC) is the sixth most common cause of cancer death and is an epidemic in the West and developed world [1, 2]. Early diagnosis improves patient prognosis, however, the disease is relatively asymptomatic in the early stages and the majority of patients presenting with symptoms are diagnosed with advanced disease. The standard of care for OAC patients in the UK is currently neoadjuvant chemotherapy followed by surgical resection [3, 4]. Although surgery is generally successful for early stage disease, and mortality rates are low, as most patients present with late stage disease surgery alone is not sufficient to prevent disease recurrence in the long-term [5]. Patients typically receive a combination of epirubicin, oxaliplatin (or cisplatin) and capecitabine (5-fluorouracil) prior to surgery, to reduce locoregional recurrence and improve patient outcome [4, 6]. However, recent trials have highlighted the benefits of neoadjuvant combined chemoradiation therapy (neo-CRT), which is already the standard of care in most other European countries [4, 7]. Presently, there are a number of on-going definitive trials directly comparing neoadjuvant chemotherapy versus combined chemoradiotherapy for OAC, such as the neo-AEGIS trial (clinicaltrials.gov NCT01726452). This and other trials come as a result of previous reports suggesting that patients who receive neo-CRT may be up to ten times more likely to achieve a complete pathological response (pCR), compared to patients who receive only neoadjuvant chemotherapy [8, 9]. Of the patients who receive neo-CRT, 18–35% have a pCR, which is a proxy for improved prognosis and a reported increase in 5-year survival rate up to 50–60% [10, 11]. Ongoing UK clinical trials are in place to assess the use of neo-CRT as the future standard of care for UK patients [6, 12].

Unfortunately, however, the majority of patients (~60–70%) do not respond to neo-CRT, and the 5-year survival rate for OAC patients after treatment is 23% [13]. Consequently, the patients who fail to respond to neo-CRT are subject to an aggressive treatment regimen from which they gain little or no benefit. Additionally, in some cases the disease progresses during the neo-CRT regimen, which reduces the success of surgery and adversely affects patient prognosis [14, 15]. The identification of biomarkers, in a pre-treatment setting, which predict patient response to neo-CRT could aid treatment stratification for patients at the point of diagnosis. Furthermore, novel therapeutics agents, targeting functional regulators associated with response to treatment, could be exploited to enhance patient response to conventional CRT as part of multimodal treatment regimens.

MicroRNAs (miRNA/miR) are a family of short non-coding RNA that repress the translation of mRNA targets [16]. Perfect Watson-Crick binding between the miRNA and its mRNA target is not essential for regulation, therefore a single miRNA can potentially target thousands of mRNA [17]. MiRNAs are predicted to regulate 30–60% of protein coding genes and are essential regulators of normal cellular processes. Approximately 50% of miRNA genes are located within cancer-associated genomic regions or chromosomal fragile sites, and are susceptible to amplification, translocation or deletion [18]. There is global downregulation of miRNA expression in cancer tissue compared to normal tissue, and dysregulated miRNA expression plays a causal role in the development and progression of cancer [10]. Cancer associated miRNAs are referred to as ‘oncomirs’, and they may act as tumour suppressors or oncogenes [19]. The link between dysregulated miRNA expression and cancer has potential clinical applications, as miRNAs are promising cancer biomarkers and novel therapeutic targets. Furthermore, miRNAs have been identified as predictors and modulators of chemo- and radiotherapy treatment sensitivity in cancer [20, 21]. Advances in miRNA profiling techniques have identified miRNA signatures predictive of treatment response by screening patient tissue samples [21]. Predictive miRNA signatures are promising clinical biomarkers, however, these miRNAs are also potential modulators of treatment response and hold greater promise as therapeutic targets. Therapeutic replacement or silencing of miRNAs that are known to modulate tumour response could improve patient response to neo-CRT, and for OAC patients would have a significant impact on outcome and survival. In this study the aim was to identify, and investigate, the role of miRNAs that potentially modulate tumour response and sensitivity to chemo- and radiotherapy in OAC.

## Materials and Methods

### Patients, treatment and histology

Ethical approval from the St. James’ Hospital/The Adelaide and Meath Hospital Dublin Institutional Research Ethics Committee (Reference 2011/27/01), and written informed consent from the patients, were obtained for this study. Pre-treatment diagnostic biopsy specimens were obtained from patients with an operable oesophageal cancer. The specimens were stored at -80°C in a biobank whilst the patients received neo-CRT and surgery. The neo-CRT treatment regimen was administered as previously described [22, 23]. Surgical resection was performed within 1 month of completing the neo-CRT regimen. The resected oesophagectomy specimens were examined by a pathologist and assigned a tumour regression grade (TRG) on a scale of 1–5, as previously described [24].

### Tissue collection

Diagnostic endoscopic biopsies were taken by a qualified endoscopist prior to neo-CRT. Specimens were stored in RNAlater (Ambion, UK) at 4°C for 24 h prior to long term storage at -80°C in the biobank [22].

### MiRNA profiling of patient tumour specimens

Total RNA was extracted from biopsy specimens and global miRNA (742) profiling arrays were performed using Human mercury LNA Universal RT miRNA arrays (Exiqon, Denmark), as previously described [22]. Analysis was performed using GenEx 5.0 software (MultiD Analyses, Life Technologies, UK) [22].

### Cell lines and cell culture

The OE33 and OE19 cell lines were purchased from the ECACC (Catalogue numbers; 96070808 and 96071721). Cells were cultured in RPMI 1640 (Lonza, Switzerland) medium supplemented with 10% foetal bovine serum (Bio-Whittaker, Lonza, Switzerland), 1% penicillin/streptomycin (Lonza, Switzerland) and 1% GlutaMAX (Invitrogen, UK) henceforth referred to as complete medium. Cells were maintained in a 37°C incubator with 95% humidified air and 5% CO_2_.

### Irradiation

X-ray irradiation was performed using an RS-2000 Pro biological research irradiator (Rad Source Technologies, Georgia, USA) at a dose rate of 1.87 Gy/min.

### Chemotherapy treatment

Solutions of *cis*-diamminedichloroplatinum (cisplatin) and 5-flurouracil (Fisher Scientific, UK) were prepared in phosphate buffered saline (Fisher Scientific, UK) and DMSO (Sigma-Aldrich, UK), respectively, and aliquoted stock solutions were stored at -20°C. Once thawed the stock solution was diluted in complete medium and applied to cells for 24 h. The IC_50_ doses for cisplatin and 5-FU were determined for the OE33 and OE19 cell lines. For the OE33 cell line 1 μM cisplatin and 12–15 μM 5-FU were used. For the OE19 cell line 3 μM cisplatin and 20 μM 5-FU were used.

### Plasmid transfection

The miRNA precursor plasmid (Catalogue number; PMIRH330PA-1) and miRZip plasmid (Catalogue number; MZIP-330-5p-PA-1) (System Biosciences, California, USA) were transfected into cells using Lipofectamine 2000 transfection reagent (Invitrogen, UK), as per the manufacturer’s instructions. These plasmids contained a GFP reporter gene and transfection efficiency was assessed by fluorescent microscopy (Olympus IX71, Hamburg, Germany) (S1 Fig.), GFP Western blotting and qPCR analysis (S2 Fig.) of miR-330 expression. The miRZip plasmids also contained the mammalian puromycin resistance gene, and stable cell lines were established after treating transfected cells with 3 μg/mL puromycin (Sigma-Aldrich, UK) over approximately 3 weeks. The miRNA precursor plasmids were employed for transient transfection, as these plasmids do not encode a mammalian selection marker. Appropriate vector control plasmids were included in all experiments; these plasmids contained a scrambled non-targeting sequence (Catalogue numbers; CD511B-1 and MZIP000-PA-1) (System Biosciences, California, USA).

### RNA extraction and quantitative PCR (qPCR)

Total RNA was extracted from cell pellets using an RNeasy mini kit (Qiagen, Netherlands). RNA was quantified using a NanoDrop ND-1000 (Thermo Scientific, UK). Reverse transcription was performed using 1 μg RNA and a QuantiTect RT kit (for mRNA) or a miScript RT kit (for miRNA) (Qiagen, Netherlands). For the qPCR 20 ng cDNA template was used with a QuantiTect SYBR Green PCR Kit (Qiagen, Netherlands) and QuantiTect Primer Assays for E2F1 and B2M (Catalogue numbers; QT00016163 and QT00088935). Thermal cycling was performed in a StepOnePlus Real Time PCR System (Applied Biosciences, UK) according to the manufacturer recommendations for QuantiTect Primer Assays (Qiagen, Netherlands). Relative E2F1 mRNA expression was determined using the 2^-ΔΔCt^ (Livak) method [25].

### Western blotting

Protein was extracted from cell pellets using RIPA lysis buffer containing commercially prepared protease and phosphatase inhibitors (Roche, UK). The BCA assay (Pierce, Thermo Scientific, UK) was used to quantify protein content, and 50 μg of protein was loaded onto a 10 or 12% SDS-PAGE gels. Electrophoretically separated proteins were transferred onto PVDF (Thermo Scientific, UK) using a wet transfer tank system (BioRad, UK). Following transfer PVDF membranes were blocked with 5% non-fat milk TBST (0.1% Tween) solution. Blots were probed for E2F1 (1:1000 dilution, KH95 mouse monoclonal, Santa Cruz Biotechnology, Texas, USA), turboGFP (1:10000 dilution, mouse monoclonal, Origene, Rockville, Maryland, USA) and the loading control β-actin (1:10000 dilution, AC-15 mouse monoclonal, Santa Cruz Biotechnology, Texas, USA). Image Lab 3.0 software (BioRad, UK) was used for densitometric analysis of western blots.

### ELISA

The levels of phosphorylated Akt were measured in protein extracts using the DuoSet p-Akt ELISA (R and D systems, UK). In a 96 well plate 100 μg protein was loaded per well and the samples were run in triplicate, according to the manufactures instructions (R and D Systems, UK), and in conjunction with a standard curve.

### Clonogenic survival assay

Cells were seeded into 6-well plates and allowed to adhere overnight. Treated cells were seeded at a density of 1000 cells per well, and mock-treated cells were seeded at a density of 500 cells per well. Cells were treated with IC_50_ doses of cisplatin or 5-FU for 24 h, or treated with 2 Gy X-ray radiation. Post-treatment cells were incubated for 8–14 day to allow colonies to form. Colonies were fixed and stained with crystal violet (70% methanol, 30% H_2_O, 0.1% w/v crystal violet) for 1 h at room temperature, followed by destaining in H_2_O. Air-dried plates were imaged and colonies were counted using a GelCount instrument (Oxford Optronics, UK). The plating efficiencies and surviving fractions were calculated as previously described [26]. Colonies that could not be accurately defined and counted using the GelCount were analysed by solubilising the crystal violet stain with a 1% SDS solution overnight at 37°C. The absorbance of the resultant crystal violet solution was measured at 590 nm using a plate reader (Biotech ELx800, UK).

### Statistics

Data are presented as the mean ± standard error of the mean (SEM) and are representative of at least three independent experiments. Statistical analysis was carried out using GraphPad InStat v3. Specific statistical tests used are disclosed in the relevant figure legends. Differences were considered to be statistically significant at *p*<0.05.

## Results

### MiR-330-5p expression is downregulated in oesophageal adenocarcinoma patients who do not respond to neo-CRT

**Fig 1:**
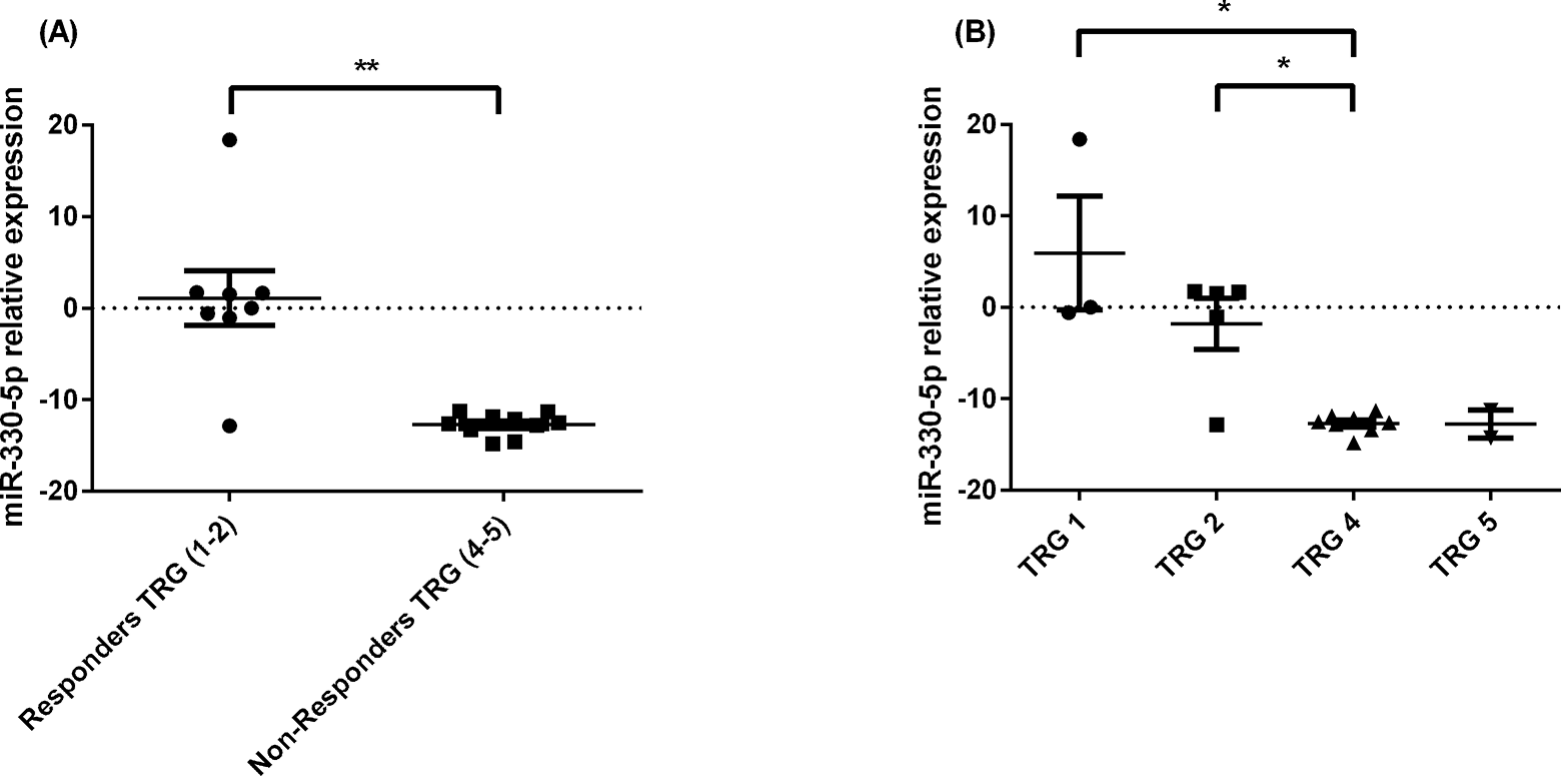
miR-330-5p expression in pre-treatment diagnostic OAC tumour biopsies from responders vs. non-responders to neo-CRT. (**A**) MiR-330-5p expression is significantly lower in patients who do not respond to neo-CRT (TRG 4 and 5) when compared with responders (TRG 1 and 2). The two outlier values in the responder data set came from the two patients who were not clinical stage TNM T3. These two outlier values were biopsy specimens derived from tumours graded T*is* and T2. Analysis was performed using the Mann Whitney U-test; **p<0.01. (**B**) MiR-330-5p expression is significantly lower in patients with TRG 4 (non-responders) compared to patients with TRG 1 and 2 (responders). Analysis was performed using the Mann Whitney U-test; *p < 0.05. Data are presented as the mean ± SEM.

In OAC patients, to identify miRNAs differentially expressed between responders and non-responders to neo-CRT, miRNA profiling arrays were used to analyse pre-treatment diagnostic biopsies. Of the 18 biopsy specimens analysed, 8 patients were responders and 10 patients were non-responders to neo-CRT. Following surgical resection the tissue removed was examined by a pathologist and assigned a TRG on a scale of 1–5. Patients categorised as TRG 1 (complete regression with no viable tumour cells evident) or TRG 2 (rare residual cancer cells) represented responders to CRT and TRG 4 (residual cancer outgrowing fibrosis) or TRG 5 (complete absence of regressive changes) represented non-responders. Patients categorised as TRG 3 were excluded for the purpose of this study. The patient cohort characteristics are outlined in Table 1. Of the 742 miRNA analysed in the array, 67 miRNAs were differentially expressed between the biopsy specimens of responders and non-responders. MiR-330-5p was the most differentially expressed miRNA between responders and non-responders, being significantly downregulated in the tumours of non-responders (Fig 1A). The expression of miR-330-5p was further assessed across the TRG groups; there was a significant downregulation in miR-330-5p expression in the TRG 4 group compared to TRG 2 group (Fig 1B). The downregulated expression of miR-330-5p in pre-treatment biopsies from non-responders, indicates a potential role in modulating targets and pathway associated with tumour response and sensitivity to the cytotoxic effects of CRT.

**Table 1:** OAC patient cohort characteristics.

Patient demographics		miRNA profiling arrays patient cohort (*n* = 18)
Gender	Male	16
	Female	2
Age (years)¹		65 (37–75)
Tumour Differentiation	Well	0
	Moderate	8
	Poor	10
Clinical TNM staging	T*is*	1
	T1	0
	T2	1
	T3	16
	T4	0
	N0	7
	N1	11
	Mx	5
	M0	13
Overall clinical TNM stage	0	0
	I	0
	IIa	6
	IIb	2
	III	10
	IV	0
TRG	1	3
	2	5
	3	0
	4	8
	5	2

*OAC*, oesophageal adenocarcinoma; *TNM*, tumour-node-metastasis clinical staging classification; *Tis*, carcinoma *in situ*; *N0*, lymph node negative; *N1*, lymph node positive; *Mx*, distant metastasis could not be evaluated; *M0*, no distant metastasis; *TRG*, tumour regression grade.

¹Values are median (range)

### MiR-330 regulates expression of the E2F1 protein and the levels of p-Akt

It has previously been demonstrated in prostate cancer that miR-330 acts as a tumour suppressor by downregulating E2F1 protein expression and the cellular levels of p-Akt, thereby promoting apoptosis of cancer cells [27]. The E2F1/p-Akt pathway has been well established to promote cell survival by inhibiting pro-apoptotic proteins that induce cell death [27–29]. Furthermore, increased levels of p-Akt are known to be induced in response to chemotherapeutics and radiation to promote cell survival and evasion of cell death [30, 31]. Based on these previous observations, it was considered that the downregulated miR-330 expression observed in the pre-treatment OAC biopsies from non-responders potentially induces the E2F1/p-Akt cell survival pathway, thereby promoting tumour resistance to CRT.

**Fig 2:**
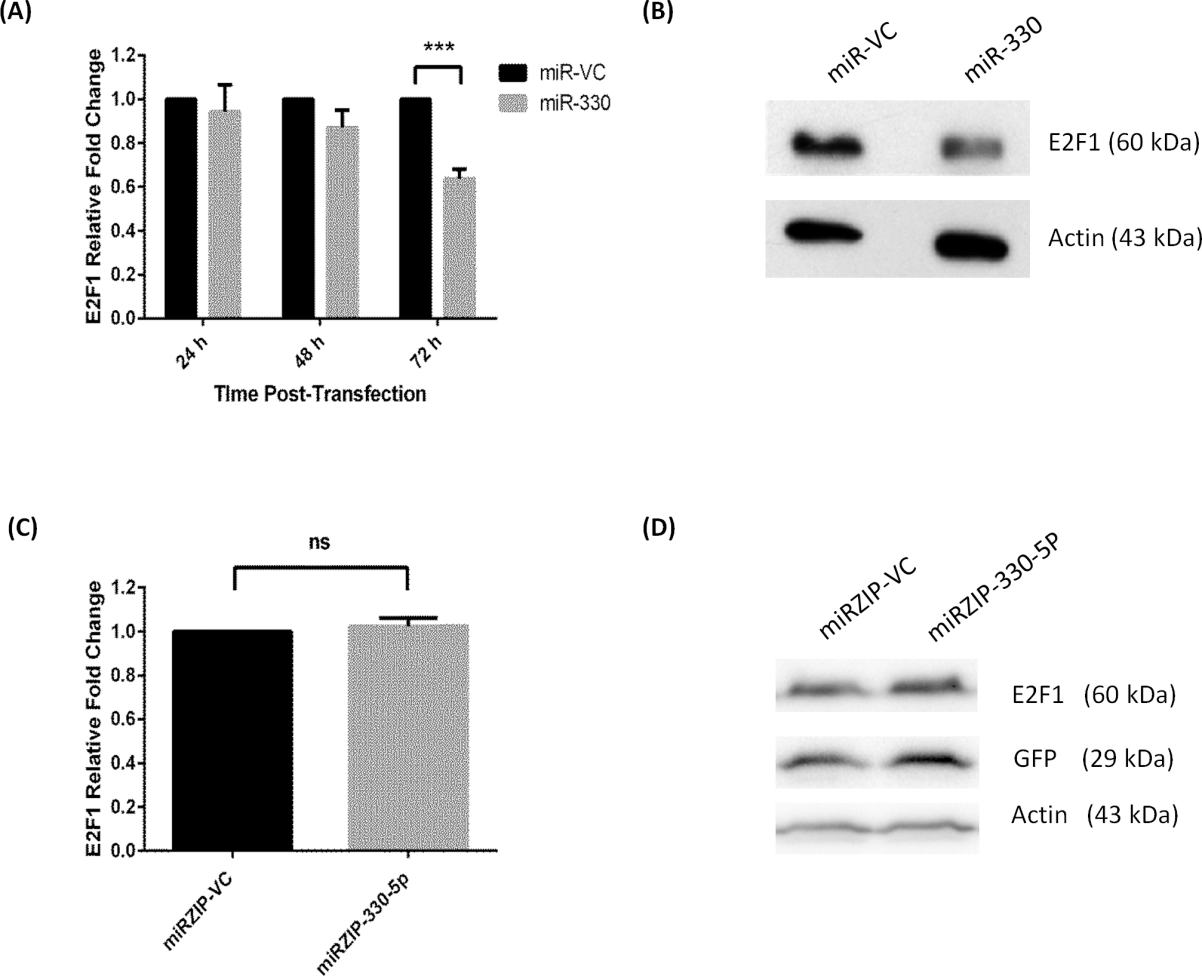
Alterations in E2F1 protein expression with miR-330 overexpression and silencing. Transient miR-330 overexpression (**A** and **B**) significantly decreased E2F1 protein expression when compared to the miR-VC (vector control). Densitometry was used to analyse western blot images. Analysis was performed using the one sample t-test; miR-VC 72 h vs. miR-330 72 h ***p < 0.001. Silencing miR-330-5p (miRZIP-330-5p) (**C** and **D**) did not alter E2F1 protein expression when compared to the miRZIP-VC (vector control). Data are presented as the mean ± SEM.

**Fig 3:**
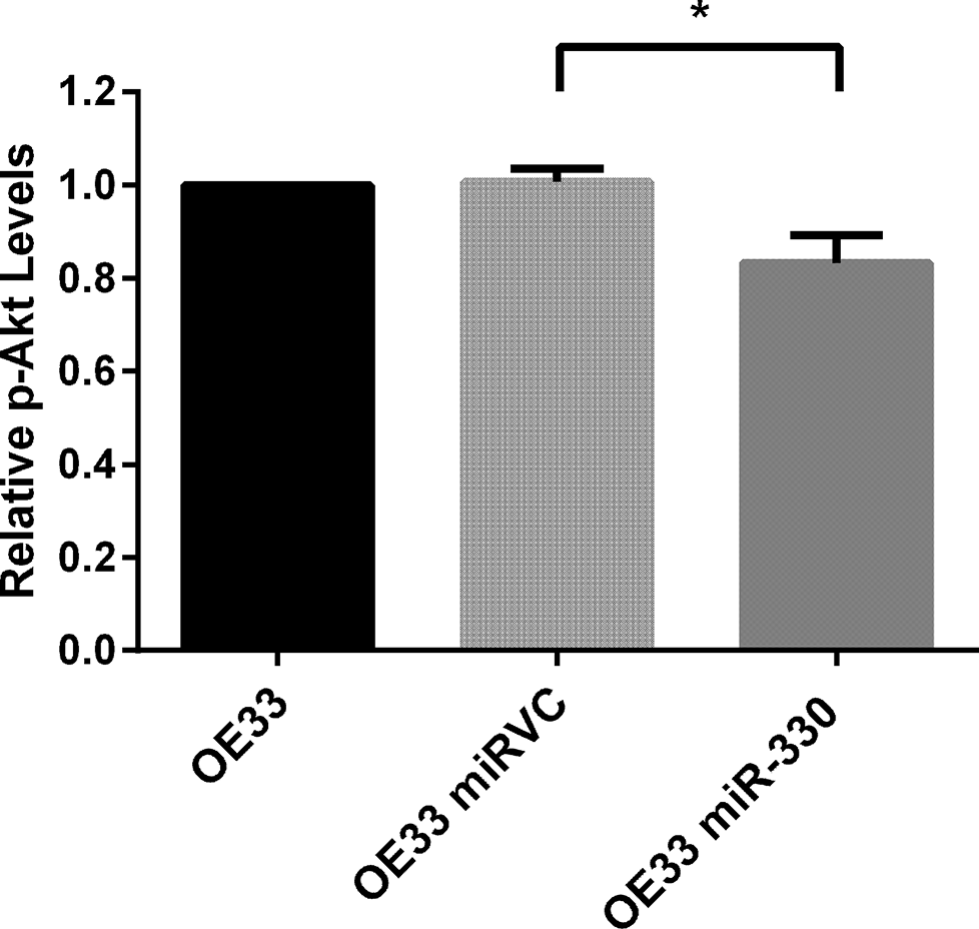
MiR-330 overexpression induces a downregulation in the levels of p-Akt. Transient miR-330 overexpression induced a decrease in the levels of p-Akt, 72 h post-transfection, in concordance with a decrease in E2F1 protein expression. Analysis was performed using one-way ANOVA and Tukey post-test; *p < 0.05. Data are presented as the mean ± SEM.

To manipulate miR-330 expression *in vitro*, OAC cell lines were transfected with a miRNA precursor construct encoding the miR-330 precursor sequence (for overexpression) or a plasmid encoding an anti-sense miR-330-5p sequence (for silencing). The overexpression plasmid construct produces both miR-330-3p and miR-330-5p. Therefore, overexpression refers to a general miR-330 overexpression, as it is not possible to discriminate between the contributions of miR-330-3p and -5p in this model. In line with the previous findings in prostate, overexpression of miR-330 significantly downregulated E2F1 protein levels (Fig 2A and 2B), and subsequently the levels of p-Akt also decreased (Fig 3) [27]. These data also confirmed the biological activity of the vector. Interestingly, E2F1 mRNA levels did not decrease with the overexpression of miR-330, suggesting that miR-330 represses post-transcriptional E2F1 mRNA translation, rather than degradation of the message (S3 Fig). The silencing vector encodes the complementary antisense sequence to miR-330-5p, which was specifically downregulated in the patient tumours. The antisense RNA produced by the plasmid binds specifically and irreversibly to endogenously expressed miR-330-5p in the cells. Silencing miR-330-5p did not alter expression of the E2F1 protein (Fig 2C and 2D).

These data indicate that miR-330 regulates, at least partially, E2F1/pAkt in OAC. The overexpression of miR-330 decreased E2F1 protein expression and p-Akt levels. In line with the data from patients, this further suggests that miR-330 alterations may confer differential sensitivity to CRT. Clonogenic survival assays were subsequently used to further investigate the role of miR-330 as a modulator of cellular sensitivity to chemo- and radio-therapy.

### MiR-330-5p silencing enhances cellular resistance to radiotherapy but not chemotherapy

The expression levels of miR-330-5p were downregulated in patient tumours that failed to respond to CRT. This indicates that miR-330-5p potentially contributes to treatment sensitivity by modulating signalling pathways associated with response to cytotoxic damage induced by CRT, such as the E2F1/p-Akt pathway. Therefore it was hypothesised that low miR-330-5p expression in patient tumours prior to treatment might enhance resistance to CRT.

**Fig 4:**
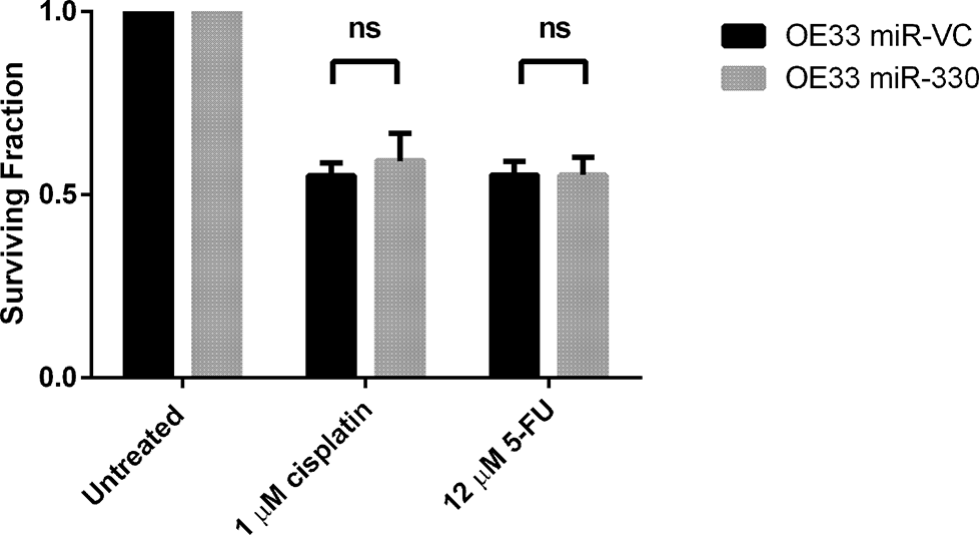
miR-330 overexpression does not alter chemosensitivity. The clonogenic survival assay was used to assess alterations in cellular sensitivity to cisplatin and 5-FU with miR-330 overexpression. The approximate IC_50_ doses of cisplatin and 5-FU were used to treat the cells for 24 h. The overexpression of miR-330 did not significantly alter cellular sensitivity to cisplatin or 5-FU. Analysis was performed using paired t-test. Data are presented as the mean ± SEM.

**Fig 5:**
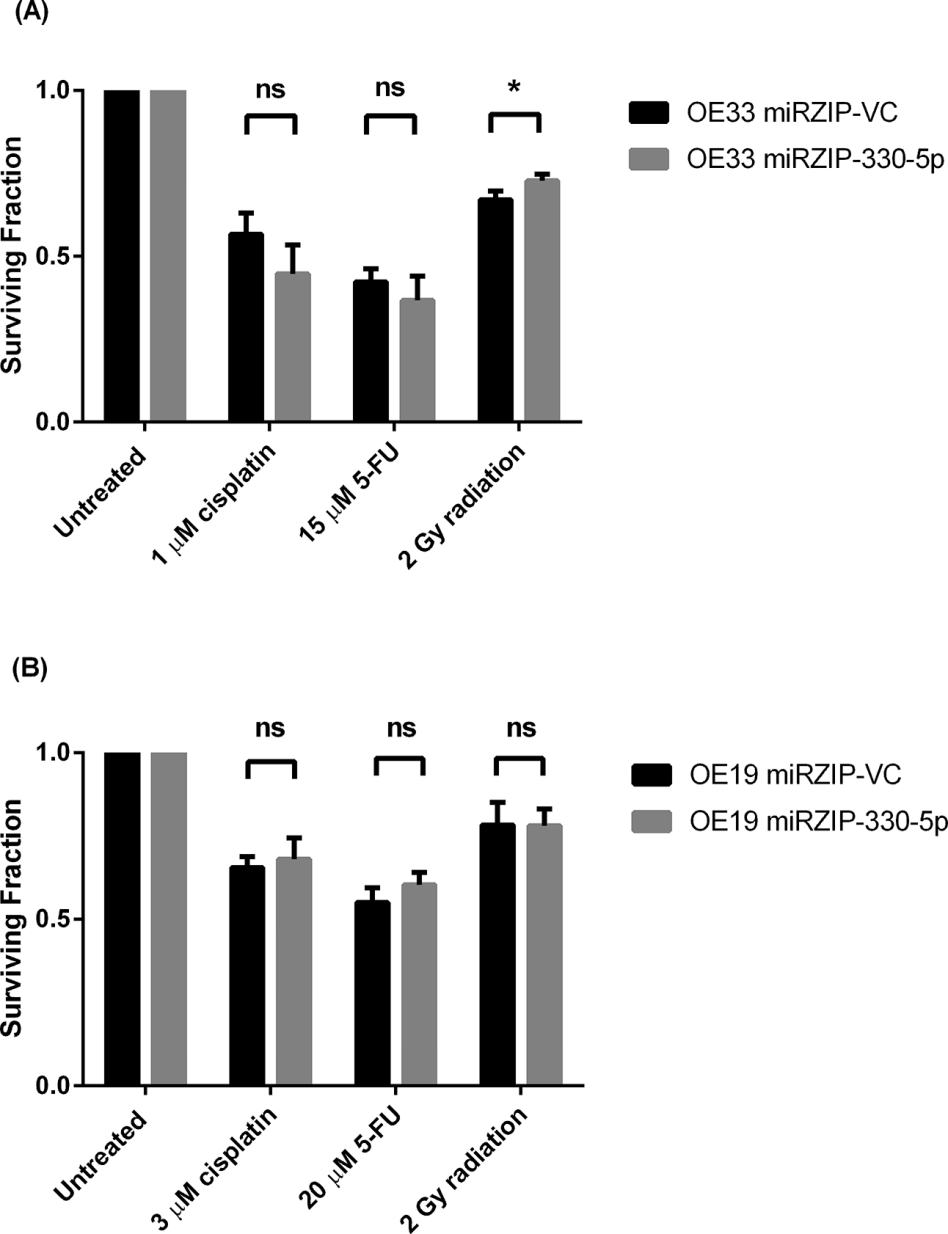
Alterations in chemo- and radiotherapy sensitivity with miR-330-5p silencing. (**A**) Silencing miR-330-5p (miRZIP-330-5p) in the OE33 cell line did not significantly alter cellular sensitivity to cisplatin or 5-FU compared to the control (miRZIP-VC). However, there was a significant increase in resistance to radiotherapy with miR-330 silencing. Analysis was performed using paired t-test; *p < 0.05. (**B**) Silencing miR-330-5p in the OE19 cell line did not significantly alter cellular sensitivity to cisplatin, 5-FU or radiation. Analysis was performed using paired t-test. Data are presented as the mean ± SEM.

The overexpression of miR-330 (both -3p and -5p) did not enhance cellular sensitivity to cisplatin or 5-FU at the selected time points and doses (Fig 4). Although alterations in E2F1 and p-Akt levels were observed with miR-330 overexpression, the negative regulation of this pathway was not sufficient to alter chemosensitivity. The silencing of miR-330-5p is a more relevant model of the downregulated expression observed in the non-responder patient biopsies, and in the OE33 cell line and a second line, OE19, miR-330-5p silencing also did not alter cellular sensitivity to cisplatin or 5-FU under the conditions tested (Fig 5A and 5B). While in the OE19 cell line miR-330-5p silencing did not enhance radioresistance (Fig 5B), the OE33 cell line was significantly more radioresistant with miR-330-5p silencing albeit marginally (OE33 miRZIP-VC 0.67 ± 0.05 *vs*. OE33 miRZIP-330-5p 0.74 ± 0.04 *p* = 0.02) (Fig 5A).

## Discussion

Currently the standard of care for OAC patients involves neo-CRT followed by surgical resection. Unfortunately, the majority of patients do not respond to treatment and the pCR rate is relatively low (25–30%) [10, 15]. The identification of biomarkers indicative of patient response to neo-CRT could aid treatment stratification in the clinic. Pre-treatment biomarkers indicative of likely therapeutic response could prevent patients who are unlikely to respond to neo-CRT receiving intensive treatment regimens, from which they will gain no benefit [15]. This approach would be particularly beneficial to those patients whose disease progresses throughout the neo-CRT regimen and for whom the delay to surgery adversely affects their prognosis. Furthermore, understanding the molecular biology of CRT resistance will help identify novel therapeutic targets as potential biological response modifiers altering tumour response to neo-CRT.

Numerous studies have identified miRNAs as potential cancer biomarkers for the purposes of diagnosis, prognosis and predicting response to treatment [20, 21]. Aside from identifying miRNA biomarkers, an understanding of the molecular biology and the functional roles of individual miRNAs is necessary for the development of novel miRNA-based therapeutics. In oesophageal cancer, specific miRNAs have been identified as potential clinical biomarkers [20, 32]. Of particular interest, in relation to this study, are those miRNAs reported to be predictive biomarkers of neo-CRT response in OAC patients [32]. Four miRNAs (miR-505-5p, miR-99b-3p, miR-451 and miR-145-3p) constitute a validated signature used to predict the response of OAC patients treated with neo-CRT [15]. The expression of these four miRNAs was used to establish a miRNA expression profile (MEP) score, which was tested in pre-treatment biopsy specimens to predict pCR in patients who received neo-CRT. The likelihood of pCR increased in a near-linear relationship with the MEP score, and identified groups of patients with high probability (≥80%) and low probability (≤10%) of pCR following neo-CRT [15]. Predictive miRNAs such as these have the potential to identify patients who are likely to respond and benefit from neo-CRT, as well as those patients who are unlikely to respond and would benefit from alternative therapies or immediate surgery. Furthermore, understanding the biological relevance of these miRNAs in the context of patient outcome, and response to neo-CRT provides an opportunity to develop novel therapeutics that could enhance sensitivity to conventional CRT, in line with our study. In previous work, using the same patient cohort and miRNA profiling presented here, miR-31 was shown to modulate tumour sensitivity to radiation [22]. Investigations into the molecular mechanism of miR-31 in OAC identified an increase in the expression of several DNA repair genes in tumours with reduced miR-31 expression [22]. The downregulated expression of miR-31 and the increase in the efficiency of the DNA repair mechanism(s) potentially contributes to resistance to radiotherapy.

While in our study time to surgery post neoadjuvant CRT was approximately 5 weeks, a recent study by the CROSS group demonstrated that increasing the time to surgery for operable OAC in neo-CRT patients from 4–6 weeks up to 12 weeks significantly improved the odds of achieving a pCR [33]. This was associated with only a slightly increased risk of postoperative complications. Based on these findings it is possible that tumours classified as non-responsive at 5 weeks may continue to regress if given additional time to surgery. Tumours classified as TRG3, which are classified as non-responsive and display histological evidence of comparable amounts of tumour and fibrotic tissue, reside on the border between the responder and non-responder classification groups. These tumours are most likely to be those re-classified with increased time to surgery, and as such were not used in our analysis.

Here, miR-330-5p downregulation was associated with poor response to neo-CRT, and a functional role of miR-330-5p as a modulator of sensitivity to CRT was investigated. For miR-330 it is not known which strand of the miRNA duplex, the -3p or -5p, is the functional mature strand, and neither strand is currently denoted as the passenger [34]. Current literature regarding miR-330 mostly reports on miR-330-3p. Reported targets of miR-330-3p in various cancer types include Sp1, CDC42 and E2F1 [27, 35, 36]. Indeed, bioinformatics identifies credible binding sites for miR-330-5p, as well as miR-330-3p, in the E2F1 mRNA sequence [37]. In prostate cancer miR-330-3p acts as a tumour suppressor by repressing the translation of E2F1 and Sp1. The downregulation of E2F1 protein levels has a downstream effect of decreasing the levels of p-Akt and induces pro-apoptotic pathways, whilst the downregulation of Sp1 protein levels has an anti-metastatic effect [27, 35]. In colorectal cancer miR-330-3p is also reported as a tumour suppressor by repressing the translation of CDC42 and negatively regulating proliferation [36]. Conversely, miR-330-3p acts an oncogenic factor in glioblastoma by enhancing proliferation, invasion and inhibiting apoptosis through activation of ERK and PI3K/AKT pathways [38, 39]. This dichotomy in the biological activities and roles of miRNAs between different cell types is well documented. To date there are no validated targets of miR-330-5p. However, a recent publication identified upregulated miR-330-5p in senescent mesenchymal stem cells; miRNAs associated with senescence have been shown to suppress regulators of the cell cycle and proliferation [40].It has previously been reported that the E2F1/p-Akt pathway promotes cell survival in response to cytotoxic insult induced by both chemotherapeutics and radiation. In prostate cancer biopsy specimens and *in vitro* culture, miR-330-3p expression was inversely correlated with expression of the E2F1 protein. The trend of miR-330-3p expression indicated a downregulation in the non-responders, although the data did not reach statistical significance.

Here, the overexpression of miR-330 downregulated E2F1 protein and the levels of p-Akt in the OE33 cell line, in line with previous literature. Silencing miR-330-5p *in vitro* was used as a model to represent the downregulated miR-330-5p expression observed in the non-responder biopsies. Silencing miR-330-5p did not alter E2F1 protein expression. To assess alterations in cellular sensitivity to chemo- and radiotherapy with miR-330 modulation, the clonogenic survival assay was employed. The clonogenic survival assay is regarded as the ‘gold standard’ for assessing cellular sensitivity to drugs and radiotherapy. While we identified no alteration in sensitivity to chemotherapeutics there was a statistically significant increase in radioresistance in the OE33 cell line with miR-330-5p silencing. The radiation dose selected for the clonogenic survival assay is clinically relevant, as part of the neo-CRT regimen patients receive radiotherapy in fractionated doses of 2–2.67 Gy [26]. Alteration in radiosensitivity was not observed in the OE19 cell line at the dose tested, and may be linked with the OE19 cell line being inherently more radioresistant than OE33 [22]. The increase in radioresistance as a result of miR-330-5p silencing *in vitro* suggests downregulated miR-330-5p in patient tumours may alter response and sensitivity to radiotherapy. Further work is needed to clarify the molecular mechanisms by which miR-330-5p modulates sensitivity to radiotherapy. Additionally, considering that patients receive daily fractioned doses of radiation, cumulatively reaching as high as 40–60 Gy, it is difficult to know how this radiosensitivity change would be represented under more translationally relevant conditions, i.e. *in vivo*.

The data presented here suggest miR-330-5p is a potential biomarker of CRT response, in a pre-treatment setting. The inclusion of miR-330-5p as part of a predictive miRNA biomarker signature could have a clinical application in stratifying treatment for OAC patients. This study is ongoing to establish the biological impact of miR-330-5p downregulation in OAC patients who do not respond to neo-CRT. It is important to recognise that miR-330-5p downregulation was originally identified in pathologically verified patient biopsies, which contain stromal-vascular cells, extracellular matrix, immune cells and regions of altered oxygen tension and vascularity. These tumour-associated compartments have been established in a variety of other models as playing an integral part in tumour biology and consequent therapeutic sensitivity. As such, the effects of miR-330-5p on tumour cell sensitivity to CRT cannot be fully explored using an *in vitro* system in the absence of other more complex aspects of the tumour microenvironment.

## Supporting Information

S1 Fig OE33 cells were transfected with the miR-330 overexpression plasmid or the miR-VC plasmid. Fluorescent microscopy shows a time course of miR-VC and miR-330 plasmid expression in OE33 cells. The plasmids contain the GFP reporter sequence. The fluorescein isothiocyanate (FITC) channel was used to acquire GFP expression images. BF, bright field; GFP, green fluorescent protein.
(TIFF)

S2 Fig qPCR validation of miR-330 overexpression relative to scrambled vector control. The graph depicts relative expression of miR-330-3p and miR-330-5p at 24 h, 48 h and 72 h post transfection. The dashed line is set a 1, and represents relative expression in the vector control at each specific time point.
(TIFF)

S3 Fig The overexpression of miR-330 does not significantly alter E2F1 mRNA levels, 72 h post-transfection. Despite a decrease in the E2F1 protein after 72 h of miR-330 overexpression the mRNA levels of E2F1 remain unchanged.
(TIFF)
