# Correction: MicroRNA-330-5p as a Putative Modulator of Neoadjuvant Chemoradiotherapy Sensitivity in Oesophageal Adenocarcinoma

**DOI:** 10.1371/journal.pone.0137155

**Published:** 2015-08-25

**Authors:** The PLOS ONE Staff

There is an error in affiliation 1 for authors Becky A. S. Bibby and Stephen G. Maher. Affiliation 1 should be: School of Biological, Biomedical and Environmental Sciences, University of Hull, Hull, United Kingdom. The publisher apologizes for the error.
