# Supplementary figures and images for: MicroRNA-330-5p as a Putative Modulator of Neoadjuvant Chemoradiotherapy Sensitivity in Oesophageal Adenocarcinoma
Source: PLoS One. 2015 Jul 29;10(7):e0134180. doi: 10.1371/journal.pone.0134180 (PMC4519309; doi:10.1371/journal.pone.0134180)

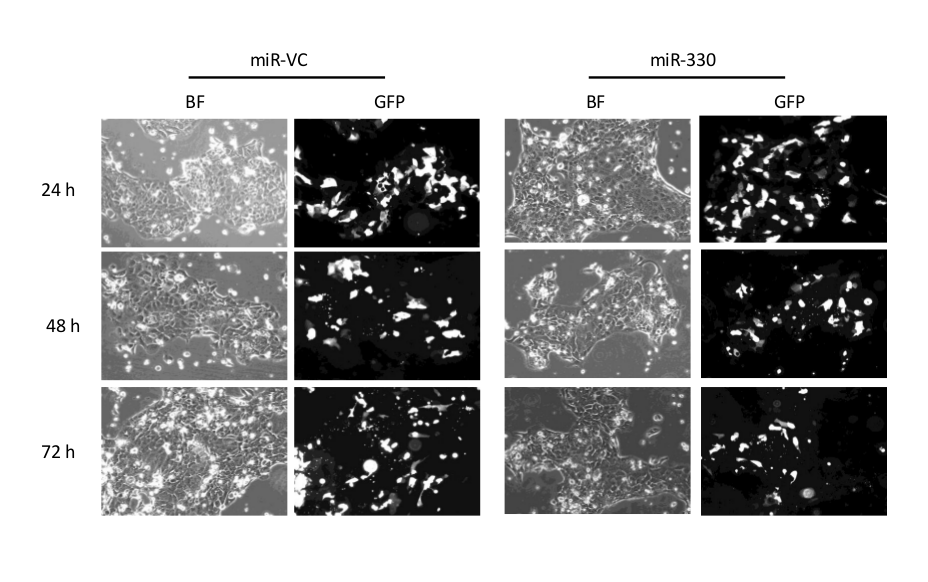

Supplement: S1 Fig — Fluorescent microscopy shows a time course of miR-VC and miR-330 plasmid expression in OE33 cells. The plasmids contain the GFP reporter sequence. The fluorescein isothiocyanate (FITC) channel was used to acquire GFP expression images. BF, bright field; GFP, green fluorescent protein. (TIFF) [file pone.0134180.s001.tiff]

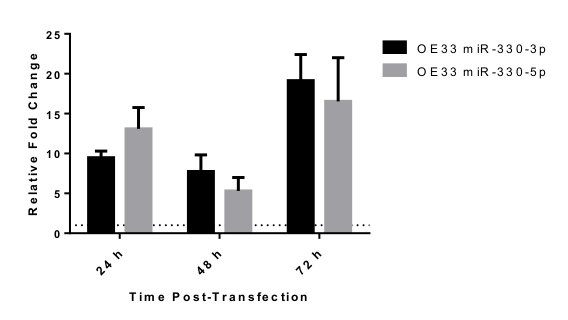

Supplement: S2 Fig — The graph depicts relative expression of miR-330-3p and miR-330-5p at 24 h, 48 h and 72 h post transfection. The dashed line is set a 1, and represents relative expression in the vector control at each specific time point. (TIFF) [file pone.0134180.s002.tiff]

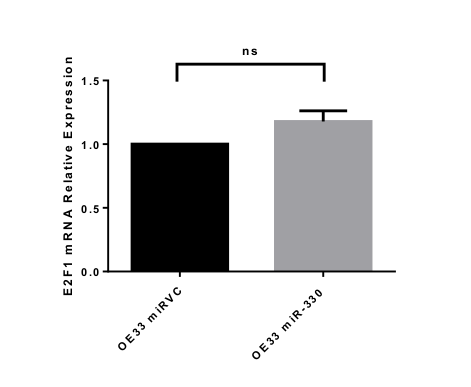

Supplement: S3 Fig — Despite a decrease in the E2F1 protein after 72 h of miR-330 overexpression the mRNA levels of E2F1 remain unchanged. (TIFF) [file pone.0134180.s003.tiff]
